# Feasibility and perception of a question prompt list in outpatient cancer care

**DOI:** 10.1186/s41687-019-0145-y

**Published:** 2019-08-15

**Authors:** Zackary Berger, Monica Tung, Pooja Yesantharao, Alice Zhou, Amanda Blackford, Thomas J. Smith, Claire Snyder

**Affiliations:** 1 Johns Hopkins University School of Medicine and Johns Hopkins Berman Institute of Bioethics, Johns Hopkins Outpatient Center, 601 N Caroline St Suite 7143, Baltimore, MD 2187 USA; 2 Johns Hopkins University School of Medicine, 733 N. Broadway, Baltimore, MD 21205 USA; 3 Johns Hopkins Division of Biostatistics and Bioinformatics, 550 N. Broadway, Suite 1111, Baltimore, MD 21205 USA; 4 Johns Hopkins University Division of General Internal Medicine and Oncology, 2024 Monument St, Baltimore, MD 21205 USA; 5 Johns Hopkins University Division of General Internal Medicine, 2024 Monument St, Baltimore, MD 21205 USA

## Abstract

**Introduction:**

Management of cancer is often characterized by difficult decisions. The National Coalition for Cancer Survivorship (NCCS) has developed the “Know Yourself” tool, a question prompt list (QPL) to enable patients to participate in these decisions.

**Methods:**

We investigated the feasibility of using the NCCS tool by oncologists and their patients with cancer in a before-and-after pilot study at a tertiary medical center. We also measured patient reported decision preparedness, anxiety, satisfaction with care, trust in physician, discussion of care with their primary care physician (PCP), and general state of health, and solicited feedback from clinicians and patients on use of the form.

**Results:**

Ninety patients and fifteen clinicians participated. Most patients reported the Tool was easy to use (91%) and would recommend it to others (73%) however fewer reported discussing the Tool at the visit (31%) or felt that it improved the quality of care (45%) or communication with the oncologist (56%). Clinicians reported Tool use in only 16 of 60 visits (27%); in these visits the Tool was helpful in identifying areas of concern (74%), guiding the clinical interaction (67%), promoting communication (62%), identifying areas of need (70%), and improving quality of care (71%). Decision preparedness, trust in physicians, uncertainty about care, anxiety, patient satisfaction and discussion of care with the PCP was unchanged with Tool use compared to non-use.

**Conclusions:**

The Know Yourself tool had poor uptake but was favorably received among both patients and clinicians who used it. These findings suggest some patients could benefit from QPLs. Future work should test how implementation strategies might achieve greater use.

## Introduction

Patients with cancer face difficult choices that require balancing competing priorities such as survival, functional capacity and symptom relief. The decisions in cancer care are often complicated by uncertainty of evidence, incomplete patient understanding and poor physician communication of the risks and benefits of treatment [1] or prognosis [2–4]. Many patients want to be engaged in their care but might depend on their physician for information and recommendations [5, 6].

A question prompt list (QPL), an intervention to encourage discussion of cancer care by doctors and patients, comprises lists of standard questions that encourage patient involvement in decision-making and offer a standard framework for patients to ask about their disease. QPLs have been found effective in the end-of-life setting. In a study of 30 patients newly diagnosed with advanced or metastatic head and neck cancer, Yeh et al. found that a QPL was well-received by patients but few used it during visits with their doctor [7]. Patients provided with a QPL asked more questions and reported fewer perceived unmet needs in a cohort of terminally ill cancer patients [8]. Rodenbach et al. found that use of a QPL increased the likelihood that cancer patients seek information (including prognosis) and express preferences about care during oncology visits [9] Despite these results, QPLs have not been widely adopted. In addition, we know of no study assessing the feasibility of a QPL used in routine outpatient oncology care.

The National Coalition for Cancer Survivorship (NCCS) Know Yourself tool (https://www.canceradvocacy.org/resources/knowyourself/) was designed to empower patients to discuss their preferences with their family and health care team in the outpatient setting. The two-page form first asks the user to consider their hopes, milestones and goals, quality of life, side effects, concerns, and support; then provides a QPL for patients to use at the clinic visit.

**Fig. 1:**
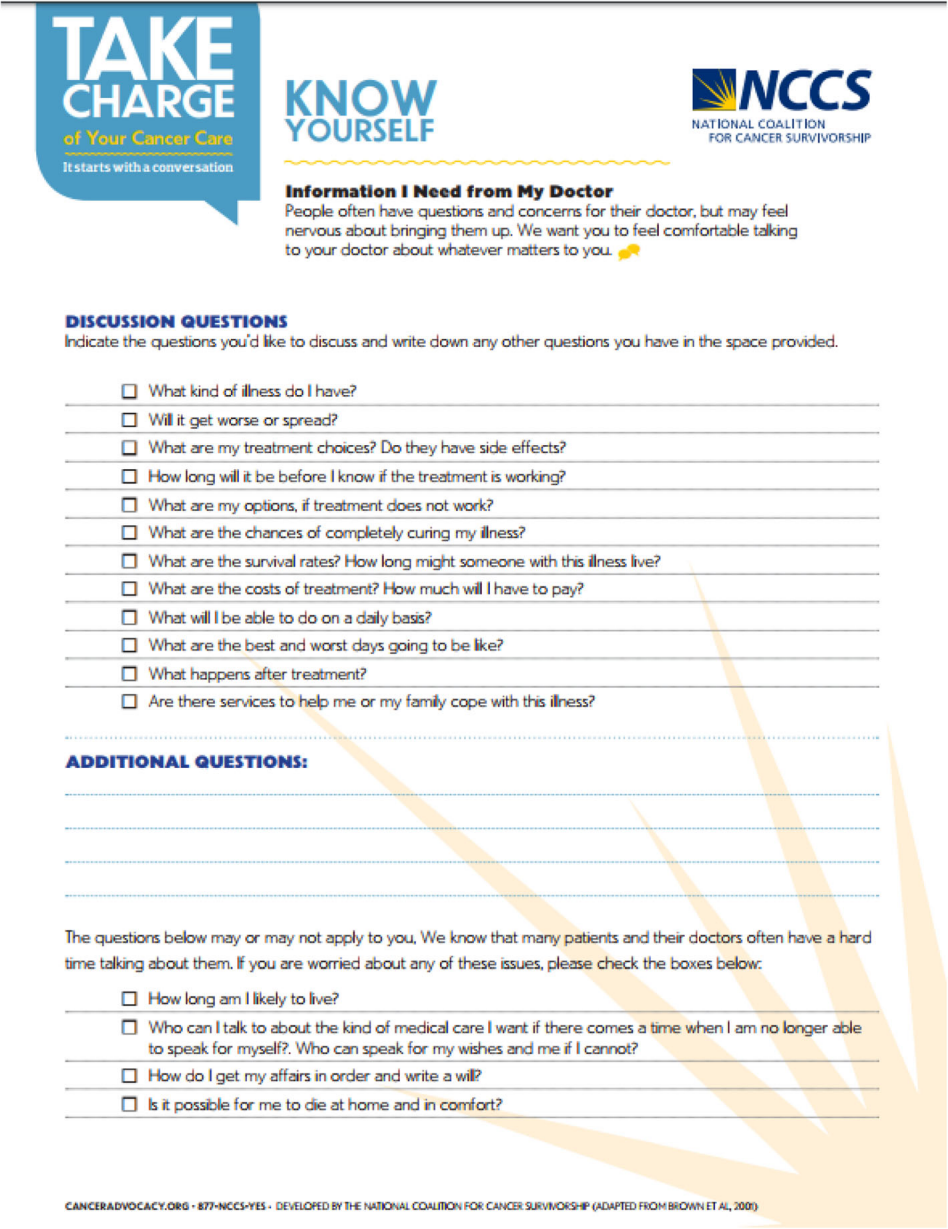
The NCCS Know Yourself question prompt list

The Tool evolved from discussions with the NCCS founder, Ellen Stovall, and Tom Smith about his “Smith Form” that he used to assist in decision making [10, 11]. This form asks the doctor to write Diagnosis, Stage, Prognosis (curable or not, and expected life span), Treatment Goals (cure, long or short term control, pain relief); specific Treatment Options; When to call the doctor; and Resuscitation Wish. The right hand column lets the doctor and patient write their responses. The NCCS, working with Tom Smith, made it into the Patient Prompt List shown in Fig. 1. It is available free of charge on the NCCS website, and is designed to be printed and discussed with the health care team [12].

This pilot study was designed to study the usability and usefulness of the NCCS tool to patients and their clinicians.

## Methods

### Study population

We used a before-and-after design in a pilot study to assess the feasibility of the NCCS tool in use by oncologists and their patients, and their experience in doing so. One medical oncologist, one radiation oncologist and one surgical oncologist were recruited for each of 5 cancer types: breast, lung, gastrointestinal, genitourinary and head and neck. For each participating clinician (*n* = 15), 6 patients were recruited: 2 for the pre-intervention group and 4 for the intervention group. These target sample sizes are consistent with previous feasibility studies) [13] Patients were eligible if they were anticipating treatment decision-making at the next visit, according to the oncologist’s judgment. The intervention group received the NCCS tool, which was described in the patient consent form as “a list of questions to help patients communicate with their cancer specialists.” The research assistant for the study contacted each participating patient before their visit with the participating provider and provided them with the form in person shortly before the visit, giving them verbal instructions for completing the form. Ethics approval was obtained from the Johns Hopkins Institutional Review Board (IRB00057116) and written informed consent was provided by each participant.

### Assessment

At enrollment, patient sociodemographic and clinical data were collected from patients and clinicians, respectively. After the decision-making visit, patient-reported data included decision preparedness (PrepDM) [14], general anxiety (GAD-7) [15], satisfaction with healthcare (PSQ-18) [16], physician trust scale [17], whether cancer care was discussed with their primary care physician and general state of health (PROMIS-SF) [18]. These are validated health status and psychosocial measures, which address outcomes important in cancer [19]. In addition, patients who had used the NCCS tool completed the Tool Feedback Form, adapted from similar studies [20]. Clinician-reported data included a practice characteristics form, knowledge of patient preferences and (in visits with intervention patients) a tool feedback form adapted from similar studies [21–23].

### Data analysis

Patient demographics were compared between pre-intervention and intervention patients with Fisher’s exact test and t tests. GAD-7, PSQ-18, physician trust, discussion of care with PCP, PROMIS-SF and physician responses on the tool feedback form were compared between pre-intervention and intervention patients’ visits using generalized linear mixed effects models, adjusting for gender, race, and medical specialty, with a random intercept for the corresponding clinician, to account for the clustering in the study design. For intervention patients, descriptive analyses summarized the PrepDM, proportion of patients completing the tool, proportion of items completed on the tool, proportion of patients and clinicians discussing the tool, and proportion of clinicians who added information to the completed tool. Analyses were conducted using R version 3.4.2 [24].

## Results

Patient and clinician baseline characteristics are found in Table 1. Of the 90 patient participants, 39 (43%) were female and 75 (83%) were white. There were no significant differences in age, gender, race, or education for the pre-intervention and intervention groups. Of the 15 clinician participants, 5 were female and 10 were white with an average 14 years in practice and median of 300 patients seen yearly.

**Table 1:** Patient and Clinician Baseline Characteristics

	Pre-Intervention Patients (*N* = 30)	Intervention Patients (*N* = 60)	*P*	Clinicians (*N* = 15)
Age - mean (SD)	63.1 (11.49)	62.75 (10.47)	0.889	
Gender - no (%)				
Male	18 (60)	33 (55)	0.822	5 (33.3)
Female	12 (40)	27 (45)		10 (66.7)
Race- no (%)				
Non-white	3 (10)	10 (17.2)	0.529	5 (33.3)
White	27 (90)	48 (82.8)		10 (66.7)
Unknown	0	2 (3.3)		
Education - no (%)				
Less than high school graduate	0	1 (1.7)	0.657	
High school graduate	5 (16.7)	6 (10)		
Some college	7 (23.3)	9 (15)		
College degree	7 (23.3)	18 (30)		
Any post-graduate work	11 (36.7)	26 (43.3)		
Cancer type - no (%)				
Breast	5 (16.7)	12 (20)	0.964	
Lung	4 (13.3)	11 (18.3)		
Gastrointestinal	8 (26.7)	15 (25)		
Genitourinary	7 (23.3)	12 (20)		
Head and neck	6 (20)	10 (16.7)		
Years in practice - median (range)				14 (2, 28)
Patients seen yearly - median (range)				300 (100, 1500)

42/60 patients in the intervention group completed the post-visit survey: 15 (36% of respondents) reported using the Tool and 13 (31%) discussing the Tool with their physician. Nearly half (49%, 18/37) reported the Tool helped them prepare for follow-up visits and know the importance of their perspective in decision making.

Patient reported use differed among cancer type. 56% of patients with head and neck cancer (5/9) reported using the tool, compared to 3 of 7 (43%) breast cancer patients, 4 of 10 (40%) genitourinary cancer patients, 2 of 8 (25%) lung cancer patients, and 1 of 8 (12%) gastrointestinal cancer patients.

Most patients reported the Tool was easy to use (91%, 32/35) and would recommend the Tool to others (73%, 24/33). Fewer patients felt that their doctor / nurse used the Tool in their care (45%, 15/33), that it improved their quality of care (47%, 15/32), made communication with the doctor easier (56%, 19/34) and made them feel in control of their care (55%, 18/33). 94% (33/35) of patients using the Tool felt that the tool’s length was just right. There was no significant difference in decision preparedness (Prep-DM), anxiety (GAD-7), patient satisfaction (PSQ-18), trust in physicians, uncertainty about cancer care or discussion of care with PCP between pre-intervention and intervention groups (Table 2).

**Table 2:** Comparison of Psychosocial and Satisfaction Outcomes in Control and Intervention Patients

	Control Patients (*n* = 30) No. Endorsing/No. Answered (%)	Intervention Patients (*n* = 60) No. Endorsing/No. Answered (%)
Clinician confidence in knowing patient’s preferences regarding clinical decisions to be made^a^	10/30 (33%)	28/59 (47%)
GAD-7 Anxiety		
Score > 10 indicating significant likelihood of anxiety disorder	3/27 (11%)	3/46 (7%)
Median Score (range)	4 (0–20)	2 (0–14)
PROMIS Short Form		
Mean (SD) Physical Health T-Score	46.04 (9.48)	47.64 (8.3)
Mean (SD) Mental Health T-Score	54.0 (8.56)	53.28 (6.97)
Patient Satisfaction (PSQ-18 subscale score > 4 indicating agreement or strong agreement that the patient was satisfied with the particular domain)		
General Satisfaction	18/27 (67%)	28/44 (64%)
Technical Quality	18/27 (67%)	30/43 (70%)
Interpersonal Manner	15/27 (56%)	31/45 (69%)
Communication	17/27 (63%)	28/45 (62%)
Financial Aspects	16/27 (59%)	21/43 (49%)
Time Spent with Doctor	15/26 (58%)	22/45 (49%)
Trust in Physician Scale		
Median Score (range)	70.45 (54.55–77.27)	70.45 (29.55–81.82)
Discussed Cancer with PCP	20/26 (77%)	25/45 (56%)
Uncertainty About Cancer Care	5/27 (19%)	3/42 (7%)

^a^Response options: very confident, somewhat confident, confident, not confident, very unsure; responses of confident, somewhat confident, or very confident counted as “endorsed”

Clinicians reported on Tool use for 16 visits. Tool was reported in a higher proportion of visits with medical oncologists (10/20, or 50%) than in visits with surgical oncologists (6/20, 30%) or radiation oncologists (0/20, 50%). Tool use was reported in 60% of visits with gastrointestinal specialists, but in no visits with lung specialists. In the 24 visits, clinicians reported the information in the Tool was helpful in identifying areas of concern (74%), guiding the clinical interaction (67%), promoting communication (62%), identifying areas of need (70%) and improving quality of care (71%). However, few clinicians noted actually using the information in the tool (27%, 16/60). A greater proportion of clinicians felt confident in knowing their patients’ preferences about decisions to be made for intervention visits (47%, 28/59) compared to pre-intervention visits (33%, 10/30), however this difference did not reach statistical significance (*p* = 0.19).

We conducted a content analysis of the hopes and concerns listed by patients who completed the Tool. We had access to 36 of 60 Tools, which were returned to us after the patient visit. The most common hope expressed by patients was for cure (*N* = 23); the most common concerns were regarding side effects (*N* = 5), emotional health (*N* = 4), and impact on family members (*N* = 4).

## Discussion

Overall, the NCCS Know Yourself tool was well received by both patients and clinicians in the minority of those who reported using it. Patients felt that the Tool positively impacted their communication with their doctor and control over their care while doctors thought the Tool helped them identify patient concerns, needs and generally promoted communication and improved quality of care. Decision preparedness, trust in physicians, uncertainty about care, and discussion of care with the PCP was unchanged with Tool use compared to non-use.

Our study was affected by certain limitations. We did not collect information on potential patients who declined to participate in the study, which might have been helpful in designing future interventions for maximum uptake. We did not collect information on tumor stage or treatment intention (curative or palliative), though it is not clear how this would have affected use of the Tool. However, certain items may have been differently perceived by patients in different clinical settings; several items might have been of greater relevance to a palliative or end-of-life setting, but may have been challenging or confronting for newly diagnosed patients or those aiming at cure.

Low uptake of the Tool (that is, its use in a minority of visits) may reflect low perceived value of the Tool, but also could result from insufficient orientation of both patients and providers regarding completion and use of the tool. This would explain the discrepancy between our findings and the study by Rodenbach et al., which included coaching from social workers regarding the use of the QPL including prioritizing their most immediate topics of interest [9]. In its original format as the “Smith Form,” it was to be started by the doctor and completed with the patient [10]. That initial endorsement by the health care team and commitment to the time and process of shared decision making may be critical to successful question prompt list use. In connection to this, another limitation of our study was that patients were provided limited information in the consent form regarding how their clinicians might use the Tool, or ways in which they could encourage use.

An explanation of the discrepancy between high ratings on the part of providers, and low use, might be social desirability bias, which might have made it more likely for clinicians to respond positively to a study conducted by their research colleagues.

The impact of the Tool on practice could have been confounded by several factors. We did not control for participants using other decision support resources during the course of the study. By giving patients the Tool immediately before the visit, we may have provided insufficient time for them to complete it. It is also possible that clinicians already elicit items included in the Tool as part of their personal practice.

As with all pilot studies, the implications of this study’s findings are primarily related to areas requiring additional investigation. In addition to testing the Tool in larger samples of patients and clinicians, future studies evaluating the use of this Tool should include greater orientation and training of both patients and providers to determine whether doing so increases uptake by patients and use by patients and clinicians during encounters. It will also be informative to identify patient, clinician and encounter factors associated with greater benefit from use of the NCCS tool; however, this pilot study examined only basic patient and clinician associations.

Patients and clinicians who used the NCCS tool found it easy to use and helpful in improving communication. Unlike decision aids, this Tool does not have treatment or disease specific information, enabling broad deployment by clinics. Building off the findings of this pilot study, future research can further elucidate whether and how this Tool helps patients prepare for different decision-making contexts and as an adjunct to specific decision aids and individualized approaches.

## Data Availability

The datasets used and/or analysed during the current study are available from the corresponding author on reasonable request.

## Abbreviations

GAD-7: Generalized Anxiety Disorder 7-item scale
NCCS: National Coalition for Cancer Survivorship
PrepDM: Patient reported decision preparedness
PSQ-18: Patient Satisfaction Questionnaire 18-item scale
QPL: Question Prompt List
